# Salvianolic Acid B Alleviates LPS-Induced Spleen Injury by Remodeling Redox Status and Suppressing NLRP3 Inflammasome

**DOI:** 10.3390/antiox14070883

**Published:** 2025-07-18

**Authors:** Hao Wang, Xiao Dou, Ruixue Wang, Yuxin Jiang, Jinsong Zhang, Xianjuan Qiao, Yingjun Liu, Hao Zhang, Chenhuan Lai, Yanan Chen, Qiang Yong

**Affiliations:** 1 National Key Laboratory for the Development and Utilization of Forest Food Resources, Nanjing Forestry University, Nanjing 210037, China; njwh1993713@163.com (H.W.); jiangyuxin@njfu.edu.cn (Y.J.); zjscjwds@njfu.edu.cn (J.Z.); lch2014@njfu.edu.cn (C.L.); swhx@njfu.com.cn (Q.Y.); 2 Key Laboratory of Veterinary Biological Products Engineering, Ministry of Agriculture and Rural Affairs, Institute of Veterinary Medicine, Jiangsu Academy of Agricultural Sciences, Nanjing 210014, China; 3 Shandong Provincial Forestry Protection and Development Service Center, Jinan 250014, China; sdsyb2019@shandong.cn (X.D.); wangruixue1115@shandong.cn (R.W.); qiaoxianjuan@shandong.cn (X.Q.); lyj@shandong.cn (Y.L.); 4 College of Animal Science & Technology, Nanjing Agricultural University, Nanjing 210095, China; h.zhang@njau.edu.cn

**Keywords:** salvianolic acid B, spleen injury, redox status, NLRP3 inflammasome, mitochondria

## Abstract

**Background**: The spleen is the primary reservoir of immune cells in mammals. Diverse stimuli can disrupt spleen homeostasis, resulting in spleen injury and immune dysfunction. This study employed a porcine model to assess the therapeutic potential of salvianolic acid B (SAB) against lipopolysaccharide (LPS)-induced splenic injury. **Methods**: Seventy-two male weanling piglets were randomly assigned to one of four groups: CON-SS, SAB-SS, CON-LPS, and SAB-LPS. The CON-SS and CON-LPS groups received a basal diet, while SAB-SS and SAB-LPS groups received a SAB-supplemented diet. After 14 d, the CON-SS and SAB-SS groups received an intraperitoneal injection of sterile saline, whereas the CON-LPS and SAB-LPS groups were injected with LPS. Blood and spleen tissues were harvested 6 h post-injection for biochemical analysis. **Results**: LPS induced systemic immune disorders in piglets, as evidenced by increased immune organ indices and decreased white blood cell, lymphocyte, and basophil counts in blood (*p* < 0.05). LPS also caused histoarchitectural disruption, cell apoptosis, oxidative stress, and inflammation in the spleen (*p* < 0.05). Conversely, SAB improved splenic histopathology and reduced splenic apoptosis and pro-inflammatory mediators in piglets (*p* < 0.05). SAB significantly mitigated peroxidation accumulation by facilitating the nuclear translocation of nuclear factor erythroid 2-related factor 2 and strengthening the antioxidant system, and inhibited nucleotide-binding oligomerization domain, leucine-rich repeat and pyrin domain-containing 3 (NLRP3) inflammasome activation (*p* < 0.05). Mechanistically, SAB attenuated LPS-induced splenic oxidative stress and NLRP3 inflammasome activation by restoring mitochondrial structure and function (*p* < 0.05). **Conclusions**: This research unveils that SAB alleviates LPS-induced spleen disorder by reinforcing antioxidant system and suppressing NLRP3 inflammasome, highlighting SAB’s potential as a prospective therapeutic agent for spleen disorders.

## 1. Introduction

The spleen, a crucial lymphoid organ, orchestrates multifaceted biological roles including hematopoiesis, blood filtration, recycling iron, and immunologic regulation [1]. As a key reservoir for immune cells, the spleen continuously monitors aging erythrocytes and immune stimulants, such as bacteria and viruses in the bloodstream, efficiently initiating both innate and adaptive immune responses to remove pathogens and cellular debris [2]. While moderate immune activation is beneficial for clearing infections, prolonged activation can lead to excessive inflammatory responses and damage the spleen function, potentially triggering immune cell exhaustion and autoimmune diseases [3]. Accumulating research has linked spleen immune dysfunction to multiple inflammatory conditions, such as sepsis [4], systemic lupus erythematosus [5], and cirrhosis accompanied by splenomegaly [6]. Thus, understanding the mechanisms of spleen immune regulation and developing strategies to maintain spleen immune balance is urgently needed.

Ample evidence has shown that immune disorder is strongly associated with oxidative stress. Immune cells located in the inflamed sites secrete copious cytokines and chemokines, that act as signaling molecules to trigger reactive oxygen species (ROS) production [7]. Surplus ROS, in turn, activate an array of transcription factors involved in inflammatory pathways, driving a detrimental feedback circuit of oxidative stress and uncontrolled inflammatory responses that culminates in cell death and tissue damage [8]. These data underscore the pivotal role of redox homeostasis in preserving splenic immunocompetence

The nucleotide-binding oligomerization domain, leucine-rich repeat and pyrin domain-containing 3 (NLRP3) inflammasome is a multiprotein platform comprising the NLRP3 scaffold, apoptosis-associated speck-like protein containing a caspase recruitment domain (ASC) adaptor, and caspase 1. This inflammasome senses pathogen- and damage—associated signaling and drives the immune process and tissue repair [9]. Upon assembly, the NLRP3 inflammasome activates caspase 1 to promote the production of key inflammatory mediators, interleukin-1β (IL-1β) and interleukin-18 (IL-18), and execute gasdermin-mediated pyroptosis [9]. Nevertheless, disrupted NLRP3 signaling further perpetuates the development and progression of splenic pathology. Guan et al. [10] have demonstrated that aberrant activation of NLRP3 inflammasome is implicated in fine particulate matter-induced spleen inflammatory injury. In addition, Zhu et al. [11] have reported that atrazine exposure leads to inappropriate NLRP3 activation, resulting in spleen injury. Although the precise mechanism behind NLRP3 inflammasome activation remains incompletely defined, mitochondrial dysfunction constitutes a pivotal contributor in this process. Mitochondrial damage brings about the accumulation of mitochondrial ROS, which oxidize mitochondrial DNA, causing it to bind and activate NLRP3 inflammasome [12]. Meanwhile, cardiolipin, a phospholipid derived from the disrupted inner mitochondrial membrane, binds to the leucine-rich repeat domain of NLRP3, recruiting it to the mitochondria and promoting inflammasome assembly [13,14]. Thus, nutritional interventions aimed at eliminating mitochondrial ROS and preserving mitochondrial structure and function may offer effective approaches for inhibiting abnormal NLRP3 inflammasome activation and restoring spleen immune balance.

Salvianolic acid B (SAB) is one of most active components of *Salvia miltiorrhiza*, formed through the condensation of three molecules of dihydroxylphenyl lactic acid and one molecule of caffeic acid. It exerts a range of pharmacological properties, particularly antioxidant and anti-inflammation activities [15,16]. During middle cerebral artery occlusion/reperfusion injury, SAB potentiates cellular ROS scavenging via upregulating nicotinamide adenine dinucleotide phosphate (NADPH) and glutathione, consequently preserving astrocyte and neuronal viability [17]. SAB also effectively inhibits NLRP3 activation in neuroinflammation model [18]. Furthermore, accumulating studies have reported that SAB improves mitochondrial disturbance by accelerating mitochondrial oxidative phosphorylation, promoting ATP production, and regulating mitophagy [19,20]. However, whether SAB confers protection against splenic immune disorders through dual modulation of redox homeostasis and NLRP3 inflammasome signaling remains unexplored.

As a potent endotoxin present in Gram-negative bacteria’s outer membrane, lipopolysaccharide (LPS) serves as a classical agent for inducing immune-mediated tissue damage in experimental systems [21,22]. Pigs, due to their anatomical, immunological, genomic, and physiological similarities to humans, are valuable models for studying human development and diseases [23]. In this study, a spleen immune disorder in pigs was established via intraperitoneal LPS injection to explore the protective potential of SAB on spleen injury and uncover the underlying mechanism. These findings will not only offer a theoretical foundation for further elucidating the biological activities of SAB, but also provide promising strategies for preventing and treating of spleen immune damage.

## 2. Material and Methods

### 2.1. Experimental Design, Management, and Sample Collection

**Figure 1:**
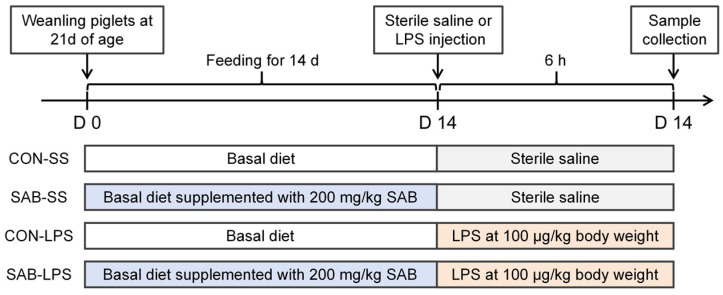
Schematic representation of the experimental procedures.

All experimental procedures involving animals strictly adhered to international ethical standards (National Research Council, 1996) and received prior approval from the Institutional Animal Care and Use Committee of Nanjing Forestry University (Permit Number 2024020, approval date 11 March 2024). A 2 × 2 factorial design within a completely randomized framework was used to evaluate the effects of SAB supplementation (0 or 200 dietary inclusion; DFT230721, ≥95% purity, Xi’an Realin Biotechnology Co., Ltd., Xi’an, China) and immunological challenge (sterile saline or LPS at 100 μg/kg body weight; 93572-42-0, Sigma-Aldrich, St. Louis, MO, USA) on piglets during a 14-day feeding trial. Seventy-two male weanling piglets (Duroc × [Landrace × Large White], 21 d of age, 7.01 kg ± 0.11 kg) were randomly allocated to one of four groups, with six replicates per group and three pigs per replicate: CON-SS, SAB-SS, CON-LPS, and SAB-LPS groups. The treatments were as follows: (1) CON-SS, piglets received a basal diet for 14 d followed by intraperitoneal injection of sterile saline; (2) SAB-SS group, piglets received a basal diet supplemented with SAB for 14 d followed by intraperitoneal injection of sterile saline; (3) CON-LPS group, piglets received a basal diet for 14 d followed by intraperitoneal injection of LPS; (4) SAB-LPS group, piglets received a basal diet supplemented with SAB for 14 d followed by intraperitoneal injection of LPS (Figure 1). LPS and SAB dosages were based on established protocols from our prior studies [24,25]. The basal diet was formulated according to the nutritional requirements for piglets (Table S1). During the trial (d 21-35 post-weaning), feed and water were offered ad libitum. The temperature in the piglet house was kept at 26–28 °C during the first week, with a gradual weekly reduction of 2 to 3 °C thereafter. Relative humidity was kept consistently 60–70%.

After 6 h of intraperitoneal injection, one piglet from each replicate (6 piglets from each treatment group and 24 piglets in total) with a body weight closest to the replicate average was selected to collect blood samples and perform routine blood marker analysis. Subsequently, the piglets were euthanized, and the spleen, thymus, and mandibular lymph nodes were excised and weighed immediately, with their relative weights calculated using the formula: relative organ weight (g/kg) = organ weight/body weight. A portion of the spleen tissue was fixed with 2.5% glutaraldehyde for ultrastructural analysis, while another portion was fixed with 4% paraformaldehyde for histopathology examination. The remaining spleen tissues were frozen in liquid nitrogen immediately.

### 2.2. Routine Blood Indicators

Peripheral blood samples collected in anticoagulant tubes containing potassium ethylenediaminetetraacetate were gently inverted 10 times immediately after venipuncture. Subsequently, routine blood indicators, including the number of white blood cells, neutrophils, lymphocytes, monocytes, eosinophils, and basophils, were determined within 1 h using a VetScan HM5 automated hematology analyzer (Abaxis, Union City, CA, USA) at room temperature. Prior to sample analysis, the instrument was calibrated with VetScan Avian/Reptilian Profile Plus controls.

### 2.3. Spleen Ultrastructure Observation

Spleen ultrastructure was observed by transmission electron microscopy (TEM). Briefly, spleen specimens were primarily fixed with 2.5% glutaraldehyde in 0.1 M sodium cacodylate buffer at 4 °C for 24 h, followed by secondary fixation with 1% osmium tetroxide in 0.1 M cacodylate buffer at 4 °C for 24 h. After three rinses with phosphate buffered saline (PBS, 10 min each), the spleen specimens were dehydrated through a graded ethanol series (50%, 70%, 90%, and 100% twice; 15 min per step), infiltrated with EPON 812 epoxy resin (propylene oxide: resin = 1:1 for 2 h, then pure resin overnight), and polymerized at 60 °C for 48 h. Ultrathin sections (60 nm thickness) were collected on 200-mesh copper grids and post-stained with 2% uranyl acetate in methanol for 10 min followed by Reynolds’ lead citrate for 5 min. Imaging was performed using a Hitachi H-7650 TEM (Tokyo, Japan).

### 2.4. Hematoxylin and Eosin (H&E) Staining

After fixation in 4% paraformaldehyde solution, spleen tissues underwent paraffin embedding via graded ethanol dehydration and xylene clearing. Serial 5-μm transverse sections were mounted on glass slides. After dewaxing and hydration, slides were treated with H&E and evaluated blindly using a Nikon ECLIPSE 80i microscopy (Tokyo, Japan).

### 2.5. Cell Apoptosis Analysis

Spleen apoptosis was quantified using a Cell Death Detection Kit (A112-02, Vazyme Biotech, Nanjing, China). Following dewaxing and rehydration, tissue sections were permeabilized with proteinase K, incubated sequentially with the TUNEL reaction mixture, and counterstained with 4′,6-diamidino-2-phenylindole (DAPI). The TUNEL-positive cells in the spleen were visualized using an Olympus fluorescent microscope (Tokyo, Japan). Apoptotic indices were calculated from 10 randomly selected high-power fields by two blinded investigators.

### 2.6. Immunofluorescent Staining

Spleen myeloperoxidase (MPO) localization was analyzed by immunofluorescence. Deparaffinized and rehydrated sections underwent antigen retrieval in a solution containing 10 mM sodium citrate. After blocking with 5% bovine serum albumin (BSA), sections were treated with rabbit anti-MPO primary antibody (ab208670, 1:200, Abcam, Cambridge, MA, USA) overnight at 4 °C, Alexa Fluor 568-conjugated goat anti-rabbit IgG (ab175471, 1:500; Abcam, Cambridge, MA, USA) for 1 h at room temperature, and DAPI. Sections were imaged using an Olympus fluorescent microscope (Tokyo, Japan).

### 2.7. Immune Status Analysis

Spleen tissues were minced, homogenized in sterile saline (1:9, wt/vol), and centrifuged at 4000× *g* for 15 min at 4 °C. The levels of tumor necrosis factor alpha (TNF-α, CSB-E16980p), interferon gamma (IFN-γ, CSB-E06794p-IS), IL-1β (CSB-E09725p), and IL-18 (CSB-E06786p) in the supernatant were determined using the enzyme-linked immunosorbent assay (ELISA) kits (CUSABIO Biotech, Wuhan, China). The protein concentrations in the homogenate were quantified using a bicinchoninic acid assay kit (P0009, Beyotime, Haimen, China). Data were normalized against total protein concentration for inter-sample comparison.

### 2.8. Redox Status Analysis

Spleen samples were homogenized in sterile saline (1:9, wt/vol) and centrifuged at 40,000× *g* for 15 min at 4 °C. The activities of catalase (CAT, A007-1-1), glutathione peroxidase (GPX, A005-1-2), and superoxide dismutase (SOD, A001-3-2), along with the contents of malondialdehyde (MDA, A003-1-2) in the homogenate, were determined using commercial kits (Nanjing Jiancheng Bioengineering Institute, Nanjing, China). 8-Hydroxy-2′-deoxyguanosine (8-OHdG, ab201734) level in the homogenate was measured with an ELISA kit (Abcam, Cambridge, MA, USA).

### 2.9. Measurement of Adenosine Triphosphate (ATP) Levels

ATP content in the spleen was determined using a luciferase-based assay (S0026, Beyotime, Haimen, China). Briefly, spleen samples were lysed and centrifuged at 12,000× *g* for 5 min at 4 °C. The supernatant was mixed with ATP detection buffer, and luminescence was measured using a Glomax20/20 luminometer (Promega, Milan, Italy). ATP levels were quantified using a standard curve generated with ATP concentrations ranging from 0 to 10 μM (0, 0.01, 0.03, 0.1, 0.3, 1, 3, 10 μM).

### 2.10. Determination of Mitochondrial Superoxide Radical Levels and Mitochondrial Complex Activities

A Mitochondria Isolation Kit (SM0020, Solarbio, Beijing, China) was used to separate mitochondria from spleen tissues. For determination of mitochondrial superoxide radical levels, mitochondrial pellets were resuspended in respiration buffer containing 10 mM succinate or 2 μM rotenone. After loading with MitoSOX Red (10 μM, 37 °C, 30 min, dark; M9940, Solarbio, Beijing, China), fluorescence was quantified using a microplate reader (ex/em = 510/580 nm; Thermo Fisher, Varioskan Flash, Waltham, MA, USA). Respiratory complex activities (BC0515, BC3230, BC3204, BC0945, BC1445) were measured using commercially available kits (Solarbio, Beijing, China).

### 2.11. Determination of Mitochondria Swelling

Mitochondria were isolated from spleen samples as described in Section 2.10. After quantification of total protein levels, mitochondrial suspension (100 μg/mL) was incubated with 1 mL of swelling assay buffer containing 150 mM KCl, 5 mM Hepes, 2 mM K_2_HPO_4_, and 5 mM glutamate, using the procedure of Du et al. [26]. Mitochondrial swelling was induced by the treatment with calcium (500 nmol/mg of protein). The absorbance at 540 nm was monitored every 60 s for 10 min using a microplate reader (Thermo Fisher, Varioskan Flash, Waltham, MA, USA), with a 20 s strong shaking interval to prevent mitochondrial deposition at the bottom of the well.

### 2.12. Evaluation of Mitochondrial Membrane Potential

Mitochondrial membrane potential was evaluated using a JC-1 Mitochondrial Membrane Potential Assay Kit (40706ES, Yeasen, Shanghai, China). Mitochondria were isolated from spleen samples as described in Section 2.10. The isolated mitochondria were incubated with JC-1 working buffer for 20 min and washed with PBS. Fluorescence intensity was detected by a microplate reader (Thermo Fisher, Varioskan Flash, Waltham, MA, USA). The mitochondrial membrane potential of spleen mitochondria was calculated based on the fluorescence ratio of JC-1 aggregates (red, ex/em = 525/590 nm) to monomers (green, ex/em = 490/530 nm).

### 2.13. RNA Isolation and Quantitative Real Time PCR (qRT-PCR) Analysis

Reagents for RNA isolation and qRT-PCR analysis were obtained from TaKaRa (Dalian, China). RNA isolation was achieved using TRIzol reagent (9108), and reverse transcription was conducted with a Prime Script RT Master Mix (6215A). qRT-PCR was executed on the ABI StepOnePlus™ Real-Time PCR system (Applied Biosystems, Foster City, CA, USA), employing SYBR Premix Ex Taq (RR390A). The relative expression of targeted genes was calculated with the 2^−ΔΔCt^ method [27]. Primer sequences are presented in Table S2.

### 2.14. Protein Extraction and Western Blot (WB) Analysis

The cytosolic and nuclear protein from spleen tissues was prepared using a Nuclear and Cytoplasmic Protein Extraction Kit (P0028), and the total protein of spleen tissues was extracted using RIPA buffer (P0013B) (Beyotime, Haimen, China). After centrifugation at 12,000× *g* for 5 min at 4 °C, equal amount of protein was loaded per lane and separated by 4–20% SDS-PAGE (ET15420Gel, ACE Biotechnology, Shanghai, China), then transferred onto polyvinylidene fluoride membranes. After blocking with 5% BSA, the membranes were incubated overnight with primary antibodies at 4 °C. Secondary antibodies were applied for 1 h at room temperature, and protein bands were visualized using an ECL chemiluminescence reagent. Details of the antibodies used are provided in Table S3.

### 2.15. Statistical Analysis

IBM SPSS Statistics 26 was used for statistical analysis. Results were analyzed by a two-way analysis of variance using the general linear model to evaluate the main effects of diet, LPS challenge, and their interaction. *p* values below 0.05 were regarded as statistically significant. When the interaction effect between diet and LPS challenge was less than 0.05, Tukey’s post hoc tests were conducted for multiple comparisons among groups. Data are shown as mean ± standard error of mean (SEM).

## 3. Results

### 3.1. Average Body Weight and Immune Organ Indices

**Figure 2:**
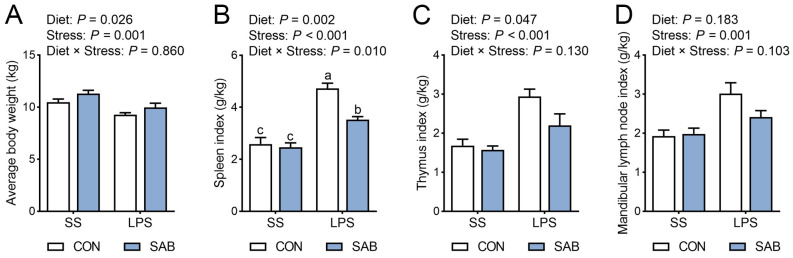
Effects of LPS and SAB on average body weight and immune organ indices of piglets. (**A**) Average body weight; (**B**) Spleen index; (**C**) Thymus index; (**D**) Mandibular lymph node index. Results are expressed as the mean ± SEM (*n* = 6). A two-way analysis of variance using the general linear model was performed to assess significant differences for diet, LPS, and their interaction. When the interaction effect was significant (*p* < 0.05), Tukey’s post hoc test was applied for multiple comparisons among groups. Columns labeled with different letters are significantly different (*p* < 0.05) based on Tukey’s test.

The LPS challenge significantly decreased the average body weight (Figure 2A) and increased the indices of the spleen (Figure 2B), thymus (Figure 2C), and mandibular lymph node (Figure 2D) in piglets (*p* < 0.05). SAB supplementation notably increased the average body weight and reduced both the spleen and thymus indices (*p* < 0.05). A significant interaction between SAB treatment and LPS challenge was found for spleen index (*p* = 0.010). Specifically, SAB-LPS piglets exhibited a lower spleen index than that of CON-LPS piglets (*p* = 0.001). These data suggest that SAB improves average body weight and counteracts the LPS-induced abnormalities in immune organ indices in piglets.

### 3.2. Blood Routine Examination

**Figure 3:**
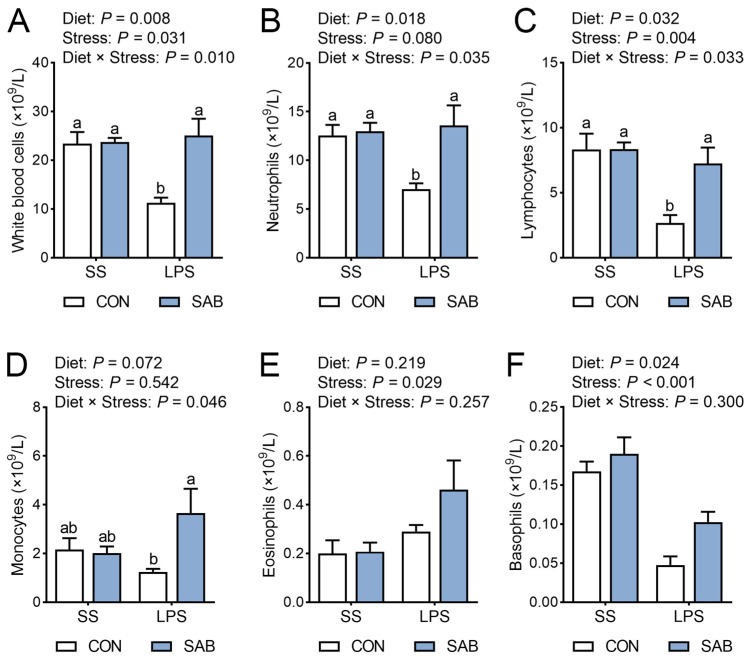
Effects of LPS and SAB on blood routine indicators in piglets. (**A**) White blood cells; (**B**) Neutrophils; (**C**) Lymphocytes; (**D**) Monocytes; (**E**) Eosinophils; (**F**) Basophils. Results are expressed as the mean ± SEM (*n* = 4). A two-way analysis of variance using the general linear model was performed to assess significant differences for diet, LPS, and their interaction. When the interaction effect was significant (*p* < 0.05), Tukey’s post hoc test was applied for multiple comparisons among groups. Columns labeled with different letters are significantly different (*p* < 0.05) based on Tukey’s test.

Changes in the lymphocyte compartment were subsequently determined by routine blood tests. The LPS-challenged piglets had lower number of white blood cells (Figure 3A), lymphocytes (Figure 3C), and basophils (Figure 3F) and higher number of eosinophils (Figure 3E) in blood, while SAB supplemented piglets had higher number of white blood cells, neutrophils (Figure 3B), lymphocytes, and basophils in blood (*p* < 0.05). There were interactions between SAB treatment and LPS challenge in white blood cell, neutrophil, lymphocyte, and monocyte counts (Figure 3D) in blood (*p* < 0.05). Compared with the CON-LPS piglets, SAB-LPS piglets exhibited increased number of white blood cells, neutrophils, lymphocytes, and monocytes in blood (*p* < 0.05). These findings indicate that SAB prevents LPS-induced blood routine alternation.

### 3.3. Histopathology and Cell Apoptosis of Spleen

**Figure 4:**
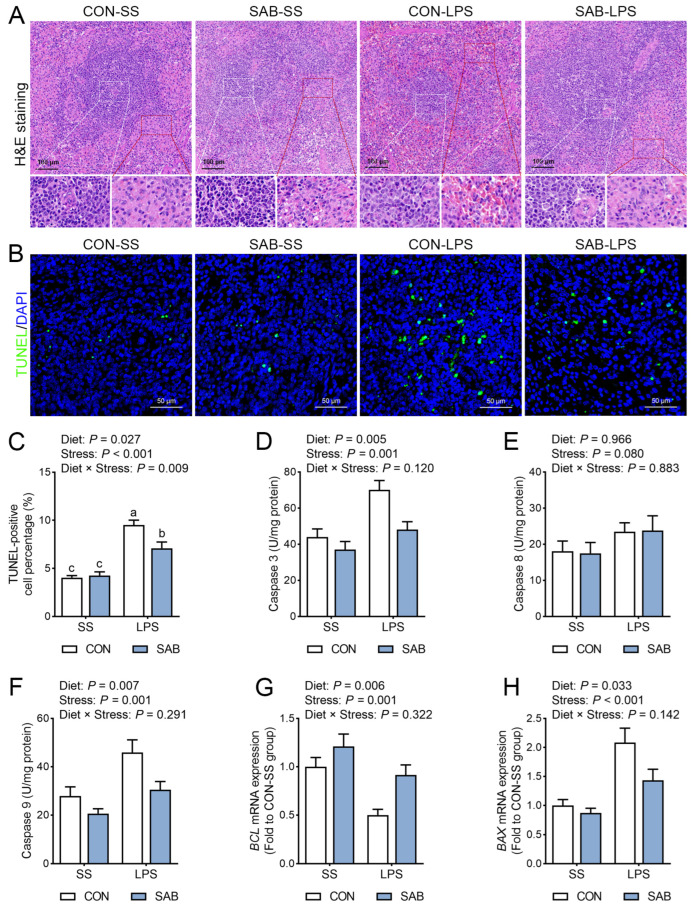
Effects of LPS and SAB on spleen histomorphology and apoptosis in piglets. (**A**) The representative images of spleen stained with H&E with a magnification of 15× and 40×, a scale bar of 100 μm, red pulp (red boxes), white pulp (white boxes). (**B**) The representative images of spleen stained with TUNEL and DAPI with a magnification of 40× and a scale bar of 50 μm. (**C**) Quantitative analysis of the TUNEL-positive cell percentage of spleen. (**D**) Splenic caspase 3 activity. (**E**) Splenic caspase 8 activity. (**F**) Splenic caspase 9 activity. (**G**) Splenic mRNA expression of *BCL2*. (**H**) Splenic mRNA expression of *BAX*. Results are expressed as the mean ± SEM (*n* = 6). A two-way analysis of variance using the general linear model was performed to assess significant differences for diet, LPS, and their interaction. When the interaction effect was significant (*p* < 0.05), Tukey’s post hoc test was applied for multiple comparisons among groups. Columns labeled with different letters are significantly different (*p* < 0.05) based on Tukey’s test.

Histological analysis of the spleen revealed that LPS challenge resulted in a blurred demarcation between the red and white pulp, white pulp expansion, and increased lymphocyte aggregation around central arteries (Figure 4A). In addition, LPS administration elevated the number of TUNEL-positive cells (Figure 4B,C), enhanced the activities of caspase 3 (Figure 4D) and caspase 9 (Figure 4F), downregulated *BCL2* mRNA expression (Figure 4G), and upregulated *BAX* mRNA expression (Figure 4H) (*p* < 0.05), indicating that LPS caused pronounced splenic apoptosis. In contrast, SAB supplementation markedly attenuated LPS-induced histopathological changes in the spleen of piglets. SAB also reduced the number of TUNEL-positive cells, suppressed the activities of caspase 3 and caspase 9, upregulated *BCL2* mRNA expression, and downregulated *BAX* mRNA expression in the spleen (*p* < 0.05). Notably, a significant interaction between SAB and LPS was observed (*p* = 0.009), with SAB-LPS piglets exhibiting fewer apoptotic cells in the spleen than CON-LPS piglets (*p* = 0.007). These findings suggest that SAB attenuates LPS-induced histoarchitectural disruption and cell apoptosis in the spleen of piglets.

### 3.4. Inflammation Response in the Spleen

**Figure 5:**
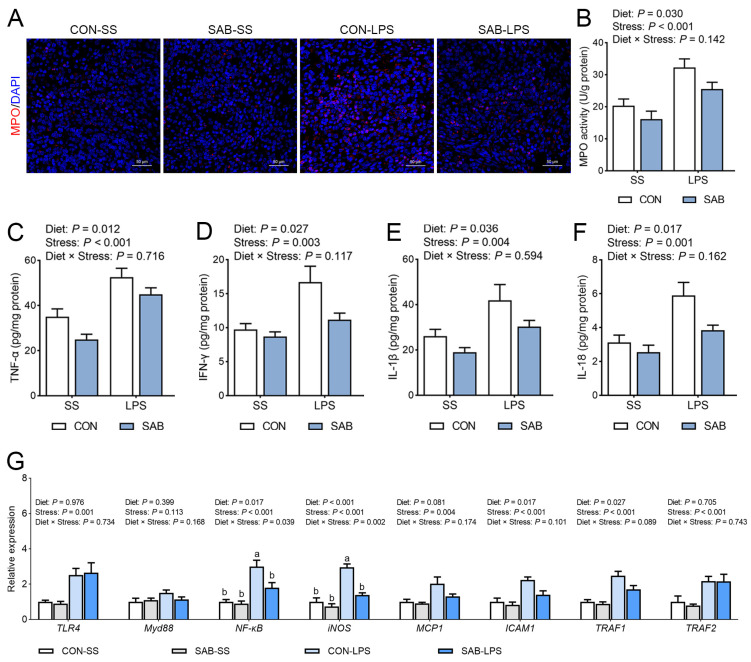
Effects of LPS and SAB on splenic immune status in piglets. (**A**) Splenic MPO expression was detected by immunofluorescence analysis and the representative images were showed with a magnification of 60× and a scale bar of 50 μm; (**B**) Splenic MPO activity; (**C**) Splenic TNF-α level; (**D**) Splenic INF-γ level; (**E**) Splenic IL-1β level; (**F**) Splenic IL-18 level; (**G**) The mRNA expression of inflammation-related genes in the spleen. Results are expressed as the mean ± SEM (*n* = 6). A two-way analysis of variance using the general linear model was performed to assess significant differences for diet, LPS and their interaction. When the interaction effect was significant (*p* < 0.05), Tukey’s post hoc test was applied for multiple comparisons among groups. Columns labeled with different letters are significantly different (*p* < 0.05) based on Tukey’s test.

To evaluate splenic immune status, we measured the protein expression and enzyme activity of MPO, along with the concentration of several inflammatory factors. Following LPS challenge, both MPO protein expression (Figure 5A) and enzyme activity (Figure 5B, *p* < 0.001) were markedly elevated in the spleen. Challenge with LPS also induced substantial increases in splenic TNF-α (Figure 5C), IFN-γ (Figure 5D), IL-1β (Figure 5E), and IL-18 (Figure 5F) levels (*p* < 0.05). In contrast, SAB treatment effectively suppressed splenic MPO protein expression and enzymatic activity, while significantly reducing TNF-α, IFN-γ, IL-1β, and IL-18 levels (*p* < 0.05). To investigate the mechanisms underlying LPS- and SAB-mediated modulation of splenic immune responses, we analyzed mRNA expression of genes related to inflammation pathway. LPS challenge markedly upregulated splenic mRNA expression of *TLR4*, *NF-κB*, *iNOS*, *MCP1*, *ICAM1*, *TRAF1*, and *TRAF2* (Figure 5G, *p* < 0.05). SAB treatment remarkably reduced splenic mRNA expression levels of *NF-κB*, *iNOS*, *ICAM1*, and *TFAF1* (*p* < 0.05). Furthermore, significant SAB × LPS interactions were observed (*p* < 0.05), with SAB-LPS piglets exhibiting lower splenic *NF-κB* and *iNOS* mRNA levels than CON-LPS piglets (*p* < 0.05). These data show that SAB inhibits LPS-mediated splenic immune disorder.

### 3.5. Redox Status of Spleen

**Figure 6:**
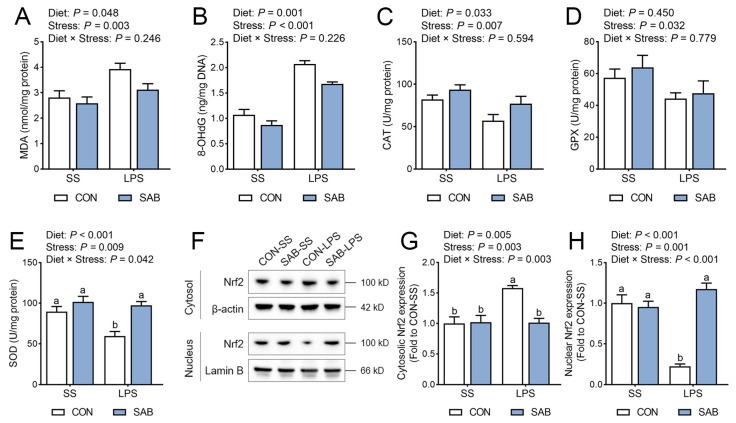
Effects of LPS and SAB on splenic redox status in piglets. (**A**) Splenic MDA content. (**B**) Splenic 8-OHdG content. (**C**) Splenic CAT activity. (**D**) Splenic GPX activity. (**E**) Splenic SOD activity. (**F**) Representative Western blot bands showing cytosolic and nuclear Nrf2 protein in the spleen. (**G**) Splenic relative expression of cytosolic Nrf2 level. (**H**) Splenic relative expression of nuclear Nrf2 level. Results are expressed as the mean ± SEM (*n* = 6). A two-way analysis of variance using the general linear model was performed to assess significant differences for diet, LPS and their interaction. When the interaction effect was significant (*p* < 0.05), Tukey’s post hoc test was applied for multiple comparisons among groups. Columns labeled with different letters are significantly different (*p* < 0.05) based on Tukey’s test.

Inflammatory processes stimulate immune cells to secrete cytokines and chemokines, which mobilize additional leukocytes to infect foci and amplify ROS production. Therefore, we next assessed splenic redox status by measuring peroxidation product contents and antioxidant enzyme activities. Our results suggested that LPS-challenged piglets exhibited elevated splenic MDA (Figure 6A) and 8-OHdG (Figure 6B) levels, alongside reduced CAT (Figure 6C), GPX (Figure 6D), and SOD (Figure 6E) activities (*p* < 0.05). SAB treatment significantly decreased MDA and 8-OHdG levels while increasing CAT and SOD activities in the spleen (*p* < 0.05). A significant LPS × SAB interaction was observed for SOD activity (*p* = 0.042), with SAB conferring substantial rescue from LPS-induced reduction in SOD activity (*p* = 0.001). Nuclear factor erythroid 2-related factor-2 (Nrf2) orchestrates cellular antioxidant responses by translocating to the nucleus and initiating transcription of cytoprotective genes such as SOD and CAT. Here, LPS exposure increased cytosolic expression levels of Nrf2 (Figure 6F,G), but reduced nuclear Nrf2 accumulation (Figure 6H) (*p* < 0.05). Conversely, SAB promoted Nrf2 translocation from cytoplasm to nucleus (*p* < 0.05). There were significant interactions between LPS and SAB in cytosol and nucleus Nrf2 expression levels (*p* < 0.05), with SAB-LPS piglets displaying higher nuclear Nrf2 levels and lower cytoplasmic Nrf2 levels than that of CON-LPS piglets (*p* < 0.05). These data demonstrate SAB’s capacity to boost splenic antioxidant activity through Nrf2 nuclear translocation.

### 3.6. NLRP3 Inflammasome Activation of Spleen

**Figure 7:**
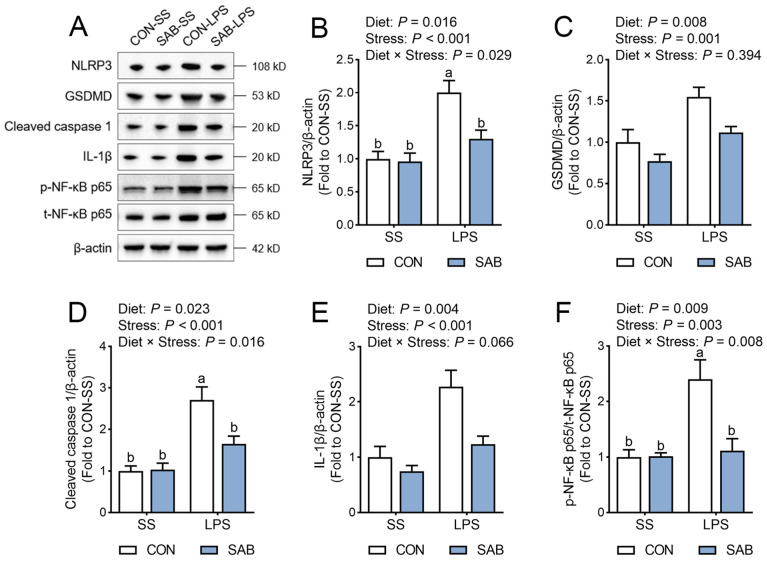
Effects of LPS and SAB on splenic NLRP3 inflammasome activation in piglets. (**A**) Representative Western blot bands showing NLRP3 inflammasome-associated pathways. (**B**) Splenic relative expression of NLRP3. (**C**) Splenic relative expression of GSDMD. (**D**) Splenic relative expression of cleaved caspase 1. (**E**) Splenic relative expression of mature IL-β. (**F**) Splenic NF-κB p65 phosphorylation. Results are expressed as the mean ± SEM (*n* = 6). A two-way analysis of variance using the general linear model was performed to assess significant differences for diet, LPS and their interaction. When the interaction effect was significant (*p* < 0.05), Tukey’s post hoc test was applied for multiple comparisons among groups. Columns labeled with different letters are significantly different (*p* < 0.05) based on Tukey’s test.

The NLRP3 inflammasome acts as a central effector of cellular inflammation, whose activation leads to tissue damage by inducing the maturation and release of pro-inflammatory mediators [28]. Here, LPS-challenged piglets exhibited higher splenic protein expression of NLRP3 (Figure 7A,B), Gasdermin D (GSDMD, Figure 7C), cleaved caspase 1 (Figure 7D), and mature IL-1β (Figure 7E), compared to their normal counterparts (*p* < 0.05). Concurrently, splenic NF-κB p65 phosphorylation (Figure 7F) was elevated after LPS exposure (*p* = 0.003). In contrast, SAB suppressed splenic protein levels of NLRP3, GSDMD, cleaved caspase 1, mature IL-1β, and phosphorylated NF-κB p65 in piglets (*p* < 0.05). Furthermore, marked interactions between SAB and LPS were observed (*p* < 0.05), with SAB-LPS piglets exhibiting lower protein levels of NLRP3, cleaved caspase 1, and phosphorylated NF-κB p65 in the spleen compared to CON-LPS piglets (*p* < 0.05). These data indicate that SAB may attenuate LPS-mediated splenic immune injury by inhibiting NLRP3 inflammasome.

### 3.7. The Ultrastructure, Swelling, and Function of Mitochondria in the Spleen

**Figure 8:**
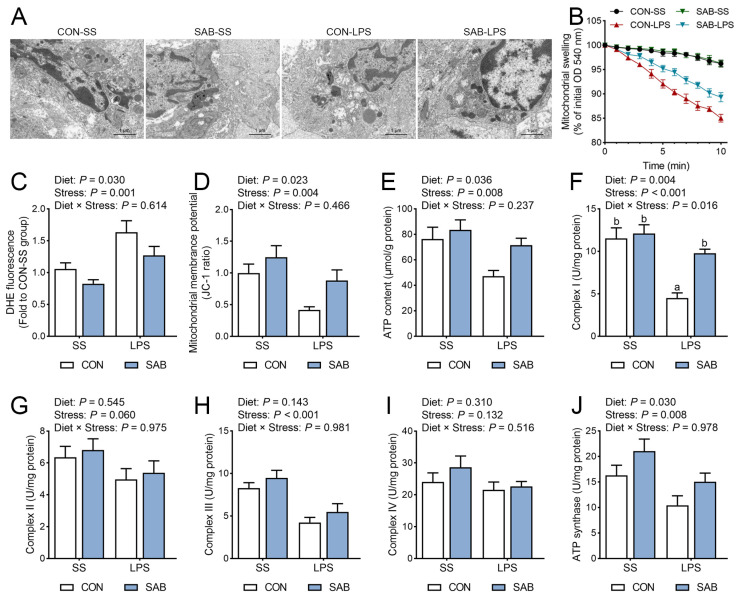
Effects of LPS and SAB on splenic mitochondrial structure and function in piglets. (**A**) Representative TEM images of spleen illustrating splenic ultrastructural features. (**B**) Spleen mitochondrial swelling was evaluated by monitoring the decreased absorbance of a mitochondrial suspension in response to calcium exposure. (**C**) Mitochondrial superoxide radical levels were detected by incubation with the DHE probe. (**D**) Mitochondrial membrane potential was evaluated using the JC-1 probe. (**E**) Splenic ATP content. (**F**) Splenic complex I activity. (**G**) Splenic complex Ⅱ activity. (**H**) Splenic complex Ⅲ activity. (**I**) Splenic complex Ⅳ activity. (**J**) Splenic ATP synthase. Results are expressed as the mean ± SEM (*n* = 6). A two-way analysis of variance using the general linear model was performed to assess significant differences for diet, LPS and their interaction. When the interaction effect was significant (*p* < 0.05), Tukey’s post hoc test was applied for multiple comparisons among groups. Columns labeled with different letters are significantly different (*p* < 0.05) based on Tukey’s test.

Mitochondria function as central hubs for both ROS production and targeting, while critically regulating NLRP3 inflammasome activation [29]. To reveal the mechanism by which LPS and SAB influence splenic redox status and NLRP3 inflammasome, several indicators associated with the mitochondrial structure and function in the spleen were assessed. TEM analysis revealed that LPS induced multiple mitochondrial lesions in splenocytes, including swelling, fragmentation, membrane integrity disruption, and mitochondrial matrix edema (Figure 8A). Subsequent assessment of mitochondrial swelling via calcium-induced absorbance reduction showed significantly greater absorbance decrease in mitochondrial suspensions from CON-LPS piglets compared to CON-SS piglets, confirming LPS-induced mitochondrial swelling (Figure 8B). Furthermore, LPS significantly increased the DHE fluorescence intensity (indicative of superoxide accumulation, Figure 8C), while decreasing mitochondrial membrane potential (Figure 8D), ATP content (Figure 8E), and complex I (Figure 8F), complex III (Figure 8H), and ATP synthase activities (Figure 8J) in the spleen (*p* < 0.05). In contrast, SAB supplementation prevented LPS-induced mitochondrial ultrastructural alterations (dilatation, cristae disorganization, and swelling). SAB also decreased DHE fluorescence intensity, elevated mitochondrial membrane potential and ATP content, and upregulated complex I and ATP synthase activities in the spleen (*p* < 0.05). Moreover, a significant LPS × SAB interaction was observed for splenic complex I activity (*p* = 0.016), with SAB-LPS piglets exhibiting higher complex I activity than CON-LPS piglets (*p* = 0.003).

## 4. Discussion

### 4.1. LPS Challenge Induces Severe Splenic Immune Injury

As the key immunological organ in mammals, the spleen is essential for both blood production and immune regulation. Dysfunction and excessive apoptosis of splenic immune cells inevitably disrupt spleen function, contributing to the occurrence and progression of systemic immune disorders [30]. In this study, LPS exposure resulted in significant abnormalities in the spleen, including increased spleen index, evident histopathological lesions, and higher apoptosis rates, which was accompanied by reduced counts of white blood cells, lymphocytes, and basophils and increased counts of eosinophils in blood. These results are consistent with the findings of Liu and colleagues [31]. Also, we observed that the spleen of LPS-challenged piglets showed higher levels of oxidative stress signs and pro-inflammation mediators. Furthermore, LPS caused a noticeable deterioration in spleen mitochondria, as indicated by apparent mitochondrial swelling, massive generation of mitochondrial superoxide radicals, together with ATP depletion and decreased respiratory complex activities. Collectively, these results suggest that LPS challenge triggers a cascade of detrimental effects on redox status, immune homeostasis, and mitochondrial function in the spleen of piglets.

### 4.2. SAB Improves LPS-Induced Splenic Inflammation by Maintaining Redox Balance

Previous studies have demonstrated that SAB exerts prominent anti-inflammatory action in diabetic nephropathy [19], inflammatory liver injury [25], and atherosclerosis [32]. Similarly, we demonstrated that SAB supplementation reduced splenic inflammation mediator levels and MPO’s expression and activity in piglets. Interestingly, administering SAB also effectively obstructed LPS-induced splenic MDA and 8-OHdG accumulation. Ample evidence has indicated that oxidative stress, characterized by an overproduction of ROS that overwhelms the antioxidant defense capacity, is a core factor in the progression of diverse inflammatory pathologies [33]. An enhanced ROS generation by polymorphonuclear neutrophils at inflammation sites aggravates inflammation response and subsequent tissue injury, whereas antioxidants can largely block these stages [34]. Actually, the antioxidant property of SAB has been well documented. Due to its nine phenolic hydroxyl groups, SAB can donate multiple hydrogen atoms, enabling it to scavenge free radicals in vivo and in vitro, effectively [15]. In addition, SAB protects against oxidative damage by modulating antioxidant defense systems through the Nrf2 signaling pathway [35,36]. In this study, SAB supplementation significantly promoted nuclear translocation of Nrf2 and upregulated SOD and CAT activities, which may represent a possible mechanism underlying the anti-inflammatory actions of SAB.

### 4.3. SAB Alleviates LPS-Induced Splenic Inflammation by Inhibiting NLRP3 Inflammasome

We also found that SAB remarkably inhibited splenic NLRP3 inflammasome activation in piglets, providing another potential explanation for the improved spleen immune status. NLRP3 is the most fully characterized inflammasome sensing threat signals, and its aberrant activation underlies many inflammatory diseases [28]. Priming and activation are the “two steps” during NLRP3 inflammasome activation. Toll-like receptors initiate priming by recognizing molecular patterns linked to pathogens or dangers and activating NF-κB, further promoting the transcription of NLRP3 and pro-IL-1β [37]. The NLRP3 inflammasome forms during the activation phase, which causes cleavage of pro-caspase 1 and maturation of IL-1β [37]. In this study, SAB supplementation downregulated NF-κB p65 phosphorylation and NLRP3 protein in the spleen, concurrently inhibiting the protein expression of cleaved caspase 1 and mature IL-1β, suggesting that SAB alleviates LPS-induced spleen inflammation response by targeting both priming and activation steps of NLRP3 inflammasome activation. Consistently, Zhao and colleagues have demonstrated that SAB reverses atherosclerosis and inflammation by suppressing nuclear translocation of NF-κB and NLRP3 inflammasome activation in TNF-α-treated human umbilical vein endothelial cells [32]. SAB has also been proposed as a promising candidate for treating Alzheimer’s diseases by regulating NLRP3 inflammasome activity and promoting microglial M2 polarization [18]. Furthermore, SAB prevents against myocardial ischemic injury by reducing NLRP3 expression and blocking caspase 1 maturation [38]. Collectively, these data demonstrate that SAB confers anti-inflammatory effect by modulating NLRP3 inflammasome activation.

### 4.4. SAB Preserves Mitochondrial Integrity to Attenuate Oxidative Stress and NLRP3 Inflammasome Activation

Mitochondrial dysfunction has been recognized as a pivotal event in both oxidative stress and NLRP3 inflammasome activation [39]. Mitochondrial dysfunction drives excessive mitochondrial ROS generation, which not only exacerbates cellular oxidative damage but also serves as a non-transcriptional priming signal for NLRP3 [40]. Meanwhile, accumulation of mitochondrial ROS facilitates oxidation of mitochondrial DNA; the resulting oxidized mitochondrial DNA directly binds to cytosolic NLRP3, triggering inflammasome assembly [12]. In a myocardial ischemia–reperfusion model, SAB effectively prevents mitochondrial ROS accumulation and subsequently NLRP3 inflammasome activation by activating sirtuin 3 [41]. In this study, SAB significantly attenuated LPS-induced mitochondrial superoxide accumulation in the spleen. This effect was likely linked to the preservation of mitochondrial ultrastructure and maintenance of mitochondrial membrane potential. Collapse of mitochondrial membrane potential potentiates ROS generation and calcium leakage, which exacerbates opening of the mitochondrial permeability transition pore and further dissipates mitochondrial membrane potential, thereby amplifying oxidative stress and cell death [39]. Notably, SAB enhanced complex I and ATP synthase activities, concomitantly increasing ATP production in LPS-challenged piglets. Improved electron transfer efficiency accelerates ATP synthesis while minimizing superoxide generation from electron leakage [29]. Thus, restoration of respiratory chain activity may underpin SAB-mediated suppression of splenic mitochondrial superoxide accumulation. Consistent with our findings, Chen et al. [42] have demonstrated that SAB prevents LPS-induced mitochondrial dysfunction by reducing mitochondrial ROS, inhibiting mitochondrial fission, and stabilizing mitochondrial membrane potential. These data suggest that SAB maintains splenic redox balance and immune homeostasis through its protection of mitochondrial structure and function.

### 4.5. The Novelty and Limitation of This Study

In this research, we demonstrated for the first time that SAB, a predominant bioactive compound in *Salvia miltiorrhiza*, effectively reversed LPS-induced systemic immune disfunction, as evidenced by reduced immune organ indices and restored peripheral immune cell counts. In addition, SAB normalized splenic histoarchitecture, inhibited pro-inflammatory mediator expression, and restored redox homeostasis in the spleen of piglets challenged with LPS. Mechanistically, SAB alleviated LPS-induced oxidative stress and NLRP3 inflammasome activation by mitigating mitochondrial swelling and superoxide overproduction while accelerating mitochondrial respiratory chain enzyme activities. These findings identified SAB as a viable candidate for preventing and treating inflammation-associated splenic injury. However, several limitations should be noted. First, although the protective action of SAB on splenic injury has been robustly demonstrated in piglets, validation in complementary models, such as rodents and organoids, remains warranted. Also, our data indicate that Nrf2 activation and NLRP3 inhibition are associated with SAB’s protection, these mechanisms need to be verified through pharmacological blockade experiments. Furthermore, future pharmacokinetic investigations, including characterization of SAB’s bioavailability, biodistribution and metabolic profile, are crucial for assessing its therapeutic potential and facilitating its clinical translation in spleen diseases.

## 5. Conclusions

This study demonstrates that SAB alleviates LPS-induced systemic immune dysfunction and splenic injury in piglets. Mechanistically, SAB’s protection is attributed to its improvement of mitochondrial structure and function, which maintains redox homeostasis and suppresses NLRP3 inflammasome activation in the LPS-challenged spleen. Further validation through pharmacological blockade and pharmacokinetic studies is required to evaluate SAB’s therapeutic potential and clinical applicability for spleen disorders.

## Data Availability

The data presented in this study are available on request from the corresponding author.

## Supplementary Materials

The following supporting information can be downloaded at: https://www.mdpi.com/article/10.3390/antiox14070883/s1, Table S1: Composition and nutrient levels of the basal diet; Table S2: Primer sequences used for quantitative real-time PCR; Table S3: Information of antibodies used for Western blot analysis.
