# Supplementary material for: Salvianolic Acid B Alleviates LPS-Induced Spleen Injury by Remodeling Redox Status and Suppressing NLRP3 Inflammasome
Source: Antioxidants (Basel). 2025 Jul 18;14(7):883. doi: 10.3390/antiox14070883 (PMC12292400; doi:10.3390/antiox14070883)
Supplement: Supplementary file 1 [file antioxidants-14-00883-s001.zip › antioxidants-3747312-supplementary.pdf]

Table S1 Composition and nutrient levels of the basal diet (%, as-fed basis unless otherwise stated)

Items	%	Nutrient levels[1]	%
Maize	62.78	Digestible energy, Mcal/kg	3.47
Soybean meal	15.00	Metabolizable energy, Mcal/kg	3.30
Fermented soybean meal	7.00	Crude protein	20.36
Extruded soybean	7.00	Total lysine	1.51
Soy protein isolate	1.30	Total methionine	0.46
Soyabean oil	2.00	Total methionine + cystine	0.86
$CaHPO_4$	1.80	Total threonine	0.94
Limestone	0.80	Total tryptophan	0.40
Salt	0.35	Total histidine	0.77
L-lysine-HCl, 78%	0.52	Total isoleucine	0.79
L-methionine	0.13	Total valine	1.20
L-threonine	0.15	Total calcium	0.82
L-isoleucine	0.10	Total phosphorus	0.65
L-tryptophan	0.01	SID[3], Methionine / Lysine	0.30
L-histidine	0.01	SID, (Methionine + Cystine) / Lysine	0.54
Calcium propionate, 50%	0.05	SID, Threonine / Lysine	0.60
Premix[2]	1.00	SID, Tryptophan / Lysine	0.26
Total	100.00	SID, Histidine / Lysine	0.49
		SID, Isoleucine / Lysine	0.52
		SID, Valine / Lysine	0.78

[1]All nutrient levels were analyzed values, except digestible energy and metabolizable energy.

[2]Provide the following per kg complete diet: Vitamin A, 8,000 IU; Vitamin D_3, 3,000 IU; Vitamin E, 20 IU; Vitamin K_3, 3 mg; Vitamin B_1, 2 mg; Vitamin B_2, 5 mg; Vitamin B_6, 7 mg; Vitamin B_{12}, 0.02 mg; Niacin, 30 mg; Pantothenic acid, 15 mg; Folic acid, 0.3 mg; Biotin, 0.08 mg; Choline chloride, 500 mg; Fe (from ferrous sulfate), 110 mg; Cu (from copper sulfate), 7 mg; Mn (from manganese sulfate), 5 mg; Zn (from zinc sulfate), 110 mg; I (from calcium iodate), 0.3 mg; Se (from sodium selenite), 0.3 mg.

[3]SID, standardized ileal digestible.

Table S2. Primer sequences used for quantitative real-time PCR

Gene name[1]	GenBank accession number	Primers sequence (5'-3')[2]	Length
TLR4	NM_001293316.1	F: TGCTTTCTCCGGGTCACTTC<br>R: TTTCACATCTGCACGCAAGG	141
Myd88	NM_001099923.1	F: CCATTCGAGATGACCCCCTG<br>R: TAGCAATGGACCAGACGCAG	183
NF-κB	NM_001114281.1	F: GGGGCGATGAGATCTTCCTG<br>R: CACGTCGGCTTGTGAAAAGG	110
iNOS	NM_001143690.1	F: GCCCAGAGGGCTTTATCACT<br>R: GCTGGGACATTTGGGGTCAT	71
MCP1	NM_214214.1	F: TCTTCAAGACCATCGCGGG<br>R: GTCCAGGTGGCTTATGGAGT	80
ICAM1	NM_213816.1	F: GAGCTGTTCAGGCAGTCAGT<br>R: GTTCACAGAAACGGGTGTGC	246
TRAF1	XM_005652719.2	F: CTGGAGATGTGGATCCTCGC<br>R: CCTGTGCAGGAAGAGAGCAA	92
TRAF2	NM_001114281.1	F: ATCGAAGCCCTGAGCAACAA<br>R: TCCAGATGAAGACGCCATCG	130
β-actin	XM_003124280.5	F: TGGAACGGTGAAGGTGACAG<br>R: CTTTTGGGAAGGCAGGGACT	176

[1]*TLR4*, toll-like receptor 4; *Myd88*, myeloid differentiation primary response 88; *NF-κB*, nuclear factor kappa B; *iNOS*, inducible nitric oxide synthase, inducible; *MCP1*, monocyte chemoattractant protein 1; *ICAM1*, intercellular adhesion molecule 1; *TRAF1*, TNF receptor-associated factor 1; *TRAF2*, TNF receptor-associated factor 2; *β-actin*, beta actin.

[2]F, forward primer; R, reverse primer.

Table S3 Information of antibodies used for Western blot analysis

Antibodies[1]	Sources	CAS number	Dilution
Nrf2	Abcam (Cambridge, MA, USA)	ab92946	1:1000
NLRP3	Proteintech (Chicago, IL, USA)	30109-1-AP	1:2000
GSDMD	Proteintech (Chicago, IL, USA)	20770-1-AP	1:5000
Caspase 1	Abcam (Cambridge, MA, USA)	ab207802	1:1000
IL-1β	Abcam (Cambridge, MA, USA)	ab283818	1:1000
p-NF-κB p65	Proteintech (Chicago, IL, USA)	82335-1-RR	1:3000
t-NF-κB p65	Proteintech (Chicago, IL, USA)	80979-1-RR	1:10000
β-actin	Proteintech (Chicago, IL, USA)	66009-1-Ig	1:20000
Lamin B1	Proteintech (Chicago, IL, USA)	12987-1-AP	1:20000
HRP-conjugated Goat Anti-Rabbit IgG(H+L)	Proteintech (Chicago, IL, USA)	SA00001-2	1:20000
RP-conjugated Goat Anti-Mouse IgG(H+L)	Proteintech (Chicago, IL, USA)	SA00001-1	1:20000

[1]Nrf2, nuclear factor erythroid 2-related factor 2; NLRP3, nucleotide-binding oligomerization domain, leucine-rich repeat and pyrin domain containing 3; GSDMD, gasdermin D; IL-1β, Interleukin-1 βeta; p-NF-κB p65, phosphorylated nuclear factor kappa B subunit p65; t-NF-κB p65: total nuclear factor kappa B subunit p65; β-actin, beta actin.
